# Deep convolutional neural network with fusion strategy for skin cancer recognition: model development and validation

**DOI:** 10.1038/s41598-023-42693-y

**Published:** 2023-10-10

**Authors:** Chao-Kuei Juan, Yu-Hao Su, Chen-Yi Wu, Chi-Shun Yang, Chung-Hao Hsu, Che-Lun Hung, Yi-Ju Chen

**Affiliations:** 1 Department of Dermatology, Taichung Veterans General Hospital, Taichung, Taiwan; 2 Department of Dermatology, National Yang Ming Chiao Tung University, Taipei, Taiwan; 3 Institute of Biomedical Informatics, National Yang Ming Chiao Tung University, Taipei, Taiwan; 4 Department of Dermatology, Taipei Veterans General Hospital, Taipei, Taiwan; 5 Department of Pathology, Taichung Veterans General Hospital, Taichung, Taiwan; 6 Department of Post-Baccalaureate Medicine, Chung-Hsing University, Taichung, Taiwan

**Keywords:** Health care, Medical research, Cancer, Skin cancer

## Abstract

We aimed to develop an accurate and efficient skin cancer classification system using deep-learning technology with a relatively small dataset of clinical images. We proposed a novel skin cancer classification method, SkinFLNet, which utilizes model fusion and lifelong learning technologies. The SkinFLNet's deep convolutional neural networks were trained using a dataset of 1215 clinical images of skin tumors diagnosed at Taichung and Taipei Veterans General Hospital between 2015 and 2020. The dataset comprised five categories: benign nevus, seborrheic keratosis, basal cell carcinoma, squamous cell carcinoma, and malignant melanoma. The SkinFLNet's performance was evaluated using 463 clinical images between January and December 2021. SkinFLNet achieved an overall classification accuracy of 85%, precision of 85%, recall of 82%, F-score of 82%, sensitivity of 82%, and specificity of 93%, outperforming other deep convolutional neural network models. We also compared SkinFLNet's performance with that of three board-certified dermatologists, and the average overall performance of SkinFLNet was comparable to, or even better than, the dermatologists. Our study presents an efficient skin cancer classification system utilizing model fusion and lifelong learning technologies that can be trained on a relatively small dataset. This system can potentially improve skin cancer screening accuracy in clinical practice.

## Introduction

Skin cancer is one of the most common cancers in Western populations, which includes malignant melanoma (Mel) and non-melanoma skin cancer (NMSC), such as basal cell carcinoma (BCC) and squamous cell carcinoma (SCC)^1,2^. Mel is responsible for most skin cancer-related deaths worldwide^1^. Early diagnosis of skin cancer is pivotal for better outcomes, boasting a 99% overall survival rate when detected earlier, however, when skin cancer spreads beyond the skin or metastasizes, the survival rate declines markedly^2–4^. Currently, dermatologists use visual inspection with the assistance of polarized light magnification via dermoscopy to examine patients. Despite their training and the use of dermoscopy, dermatologists rarely achieve diagnosis accuracy or sensitivities greater than 80% without pathologic support^5^. Medical diagnosis depends on various factors, such as the patient's history, ethnicity, social habits, and exposure to the sun. Suspicious lesions are biopsied in an office setting and sent to the laboratory, where they are processed and examined by a pathologist to render a diagnosis.

Convolutional Neural Networks (CNNs) models have demonstrated remarkable efficiency, accuracy, and reliability in image classification tasks, achieving near-human performance levels in many challenging image stratification tasks^6–10^. Additionally, CNNs have been successfully utilized in the medical field to classify diseases from medical images^11,12^. In 2017, Esteva et al.^13^ first reported a deep-learning convolutional neural network (DCNN) image classifier that performed as well as 21 board-certified dermatologists in identifying images with malignant lesions. The DCNN was trained on clinical and dermoscopic images of skin lesions and generated its diagnostic criteria for melanoma detection. Subsequent publications have demonstrated similar results, with DCNNs achieving dermatologist-level skin cancer classification^14–16^. However, it is worth noting that most studies in this area have focused on testing only two critical binary classifications, such as benign nevus and Mel.

The current skin disease datasets are biased toward fair-skinned individuals, with fewer cases from brown or dark-skinned people^17^. People with darker skin have a lower risk of skin cancer than the fair-skinned population, but they are often diagnosed at later stages^18^. Therefore, deep learning frameworks validated using fair-skinned populations may not accurately diagnose skin cancer in people with darker skin, leading to misdiagnosis and vice versa^19^. This underscores the need for more diverse datasets that represent different skin types to improve the accuracy of deep learning algorithms in diagnosing skin diseases across all populations.

While machine learning methods have made significant improvements in various applications, no single algorithm can outperform all other machine learning algorithms across all applications. To improve prediction and classification tasks, ensemble learning techniques have emerged as an effective approach, which involves creating and combining multiple models^20,21^. This approach differs from conventional machine learning techniques that train a single model using training data^22,23^. Ensemble learning algorithms can enhance the accuracy of prediction results and reduce the overfitting problem by combining the contributions of multiple models^21–24^.

Unlike human dermatologists, who can continually improve their learned skills through clinical practice, most machine learning algorithms, such as neural networks, have fixed parameters once the training process is complete, limiting their flexibility. This inflexibility poses a challenge in real-world applications as a model with fixed parameters may not be adequate for handling a variety of unseen data. Lifelong learning techniques address this challenge by enabling trained models to learn sequentially without requiring the re-training of all data again.

In this study, we proposed a novel system that combines fusion strategy and lifelong learning technologies to improve skin cancer classification accuracy. Our approach leverages the fusion strategy to achieve better classification accuracy by combining the predictions of two CNN models instead of relying on a single CNN model. We also utilized lifelong learning to train an updated model using misclassified images, which is crucial for improving the model's accuracy continually, making it suitable for clinical practice. Our findings demonstrate that using less than 2000 clinical images for DCNN training in the Fitzpatrick skin type III-IV population in Taiwan, our DCNN model performed as well as dermatologists in skin cancer classification.

## Results

### Dataset

The study was conducted at the Department of Dermatology of the Taichung Veterans General Hospital, and it was approved by the institutional review board (no. CE21044A-1). The study involved digital clinical images of skin tumors diagnosed between 2015 and 2020. The demographic information was collected. The images were extracted from the hospital's database, and only images with the following diagnoses were included: Malignant epithelial tumors (BCC and SCC), Malignant melanoma (Mel), Benign epithelial tumors (seborrheic keratosis, SK), and Benign melanocytic tumors (Nevus).

All clinical images were taken using digital cameras with at least 8 million pixels, a macro lens, and a macro ring flash. Dermoscopic images were not included in the study. All diagnoses were based on pathological examination. A total of 2078 images were included in the study, and the number of images in each classification is listed in Table 1.

**Table 1:** Skin cancer training dataset.

Skin tumor types	Training, number of images			Testing, N, images
	Original	Augmentation	Validation	
Melanoma	145	55	80	12
Malignant epithelial (BCC)	200	200	80	74
Malignant epithelial (SCC)	120	280	80	26
Benign melanocytic (Nevi)	400	0	80	287
Benign epithelial (SK)	350	50	80	64

*BCC* basal cell carcinoma, *SCC* squamous cell carcinoma, *SK* seborrheic keratosis.

Of the 2078 images, 75.2% (1215 images) were used for the DCNN training, and 18.8% (400 images) were allocated for validation. To address the class imbalance, 55, 200, 280, and 50 images of Mel, BCC, SCC, and seborrheic keratosis were included for augmentation. The images were divided by the person to prevent images from the same patient from being used in both training and testing.

### Performance of the deep convolutional neural network

**Figure 1:**
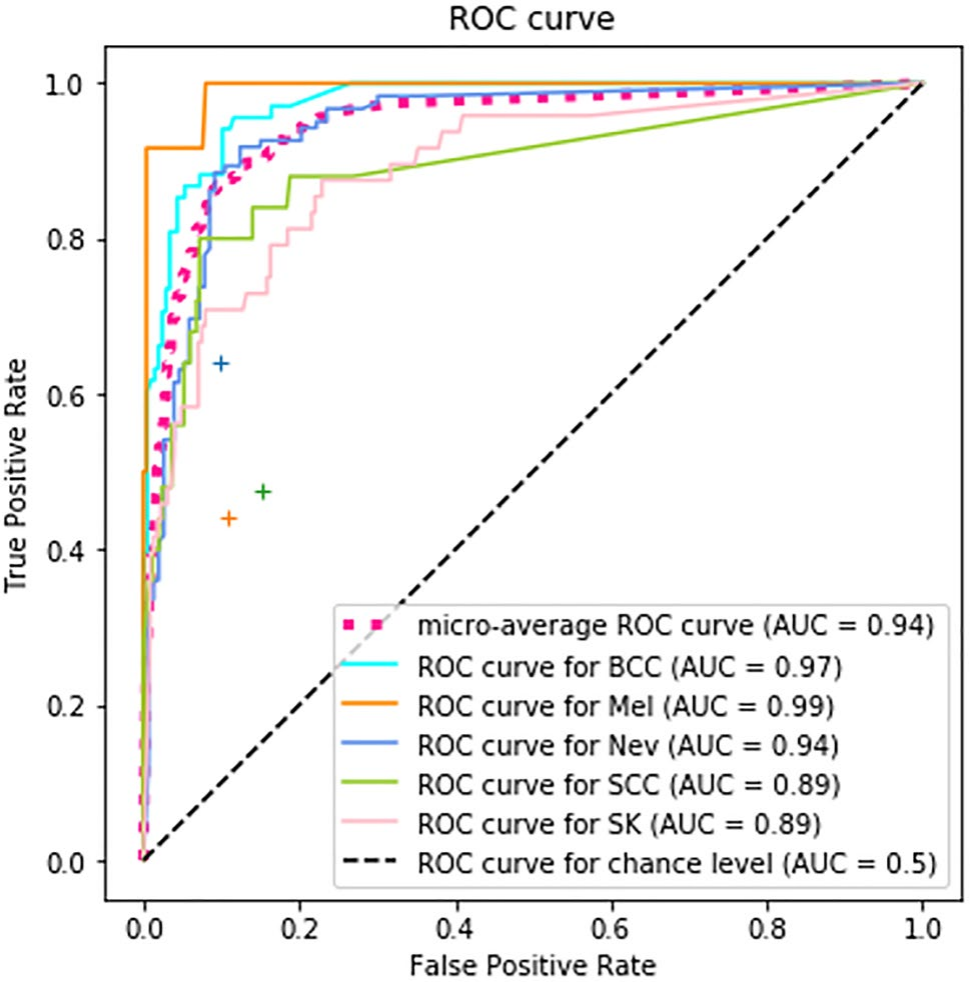
The micro-average ROC curves of SkinFLNet. The evaluation results of three dermatologists are plotted with their average performance (green and orange cross symbols).

The study utilized a diagnostic algorithm of DCNN to output the probability of different skin tumors based on clinical images, as shown in Fig. 1. The most likely diagnosis is presented as the final diagnosis. To evaluate the performance of the DCNN system, 463 clinical images of skin tumors from 270 patients who visited the dermatologic outpatient department at Taichung Veterans General Hospital and had skin tumors biopsy or excision performed between January 1 and December 1, 2021, were used. The performance of the seven trained DCNN models was compared, and the results are shown in Table 2.

**Table 2:** Performance of the deep convolutional neural networks.

	WP	WR	WF	WS	WSP
ResNet50	0.84	0.79	0.81	0.79	0.93
InceptionResNetV2	0.83	0.78	0.80	0.78	0.92
InceptionV3	0.83	0.76	0.80	0.76	0.93
VGG16	0.85	0.75	0.80	0.75	0.94
VGG19	0.82	0.73	0.77	0.73	0.93
MobileNet	0.84	0.78	0.81	0.78	0.94
MobileNetV2	0.83	0.78	0.80	0.78	0.9

*WP* weight precision, *WR* weight recall, *WF* weight F-score, *WS* weight sensitivity, *WSP* weight specificity.

According to the results, the ResNet50 model had the best overall diagnostic accuracies for weight precision (WP) (0.84), weight recall (WR) (0.79), weight F-score (WF) (0.81), weight sensitivity (WS) (0.79), and weight specificity (WSP) (0.93). It is important to note that the evaluation was based on a limited dataset, and further studies are required to validate the results.

### Performance of model fusion

In the study, seven DCNN algorithms were used to produce 21 combinations to evaluate the performance of different combinations in SkinFLNet. The performance of all 21 combinations is shown in Table 3. The combination of InceptionV3 and ResNet50 achieved the best WP and WSP. Meanwhile, the combination, InceptionResNetV2 and MobileNet achieved the best WR, WF, and WS.

**Table 3:** Performance of model fusion with different combinations in SkinFLNet.

	WP	WR	WF	WS	WSP
InceptionResNetV2 + InceptionV3	0.85	0.82	0.82	0.82	0.93
InceptionResNetV2 + ResNet50	0.85	0.82	0.82	0.82	0.93
InceptionResNetV2 + VGG16	0.82	0.80	0.80	0.80	0.91
InceptionResNetV2 + VGG19	0.84	0.79	0.79	0.79	0.93
InceptionResNetV2 + MobileNet	0.84	0.83	0.83	0.83	0.93
InceptionResNetV2 + MobileNetV2	0.84	0.80	0.80	0.80	0.93
**InceptionV3 + Res50**	**0.85**	**0.82**	**0.82**	**0.82**	**0.93**
InceptionV3 + VGG16	0.84	0.82	0.82	0.82	0.93
InceptionV3 + VGG19	0.85	0.79	0.79	0.79	0.94
InceptionV3 + MobileNet	0.84	0.81	0.81	0.81	0.93
InceptionV3 + MobileNetV2	0.85	0.80	0.80	0.80	0.93
ResNet50 + VGG16	0.83	0.81	0.81	0.81	0.93
ResNet50 + VGG19	0.86	0.81	0.81	0.81	0.94
ResNet50 + MobileNet	0.84	0.81	0.81	0.81	0.93
ResNet50 + MobileNetV2	0.86	0.82	0.82	0.82	0.93
VGG16 + VGG19	0.83	0.77	0.77	0.77	0.94
VGG16 + MobileNet	0.84	0.80	0.80	0.80	0.93
VGG16 + MobileNetV2	0.84	0.80	0.80	0.80	0.93
VGG19 + MobileNet	0.85	0.79	0.79	0.79	0.94
VGG19 + MobileNetV2	0.85	0.78	0.78	0.78	0.94
MobileNet + MobileNetV2	0.85	0.78	0.78	0.78	0.94

*WP* weight precision, *WR* weight recall, *WF* weight F-score, *WS* weight sensitivity, *WSP* weight specificity.

Significant values are in bold.

Table 4 compares the performance of the proposed model fusion algorithm and the individual DCNN algorithms. The proposed fusion model outperformed other DCNN algorithms, indicating that combining multiple DCNN algorithms can improve the accuracy of skin tumor classification.

**Table 4:** Performance of comparison among SkinFLNet and deep convolutional neural network models.

	WP	WR	WF	WS	WSP
ResNet50	0.84	0.79	0.81	0.79	0.93
InceptionResNetV2	0.83	0.78	0.80	0.78	0.92
InceptionV3	0.83	0.76	0.80	0.76	0.93
VGG16	0.85	0.75	0.80	0.75	0.94
VGG19	0.82	0.73	0.77	0.73	0.93
MobileNet	0.84	0.78	0.81	0.78	0.94
MobileNetV2	0.83	0.78	0.80	0.78	0.9
SkinFLNet	**0.85**	**0.82**	**0.82**	**0.82**	**0.93**

*WP* weight precision, *WR* weight recall, *WF* weight F-score, *WS* weight sensitivity, *WSP* weight specificity.

Significant values are in bold.

### Performance of lifelong learning

To evaluate the performance of continuous learning of SkinFLNet, a dataset of 240 images was used for continuous training, including 80 images for tumors of Mel, BCC, and SCC (MBS), 80 for Nev, and 80 for SK. Of these images, 50% were randomly selected from the original dataset, and the rest were unseen data. A test dataset of 48 images was used, including ten images for MBS, 15 for Nev, and 13 for SK.

Table 5 shows the performance metrics of the classification results by SkinFLNet before and after lifelong learning. The lifelong learning algorithm achieved WP, WR, WF, WS, and WSP scores of MBS, SK, and Nevus of 0.89, 0.90, 0.90, 0.90, and 0.92, respectively. Therefore, SkinFLNet is suitable for clinical practice to improve classification accuracy by adjusting the weights of the CNN models used in SkinFLNet.

**Table 5:** Performance of comparison of SkinFLNet before and after lifelong learning.

	WP	WR	WF	WS	WSP
SkinFLNet (before lifelong learning)	0.81	0.75	0.78	0.75	0.85
SkinFLNet (after lifelong learning)	0.89	0.90	0.90	0.90	0.92

*WP* weight precision, *WR* weight recall, *WF* weight F-score, *WS* weight sensitivity, *WSP* weight specificity.

### Performance comparison between SkinFLNet and dermatologists

**Figure 2:**
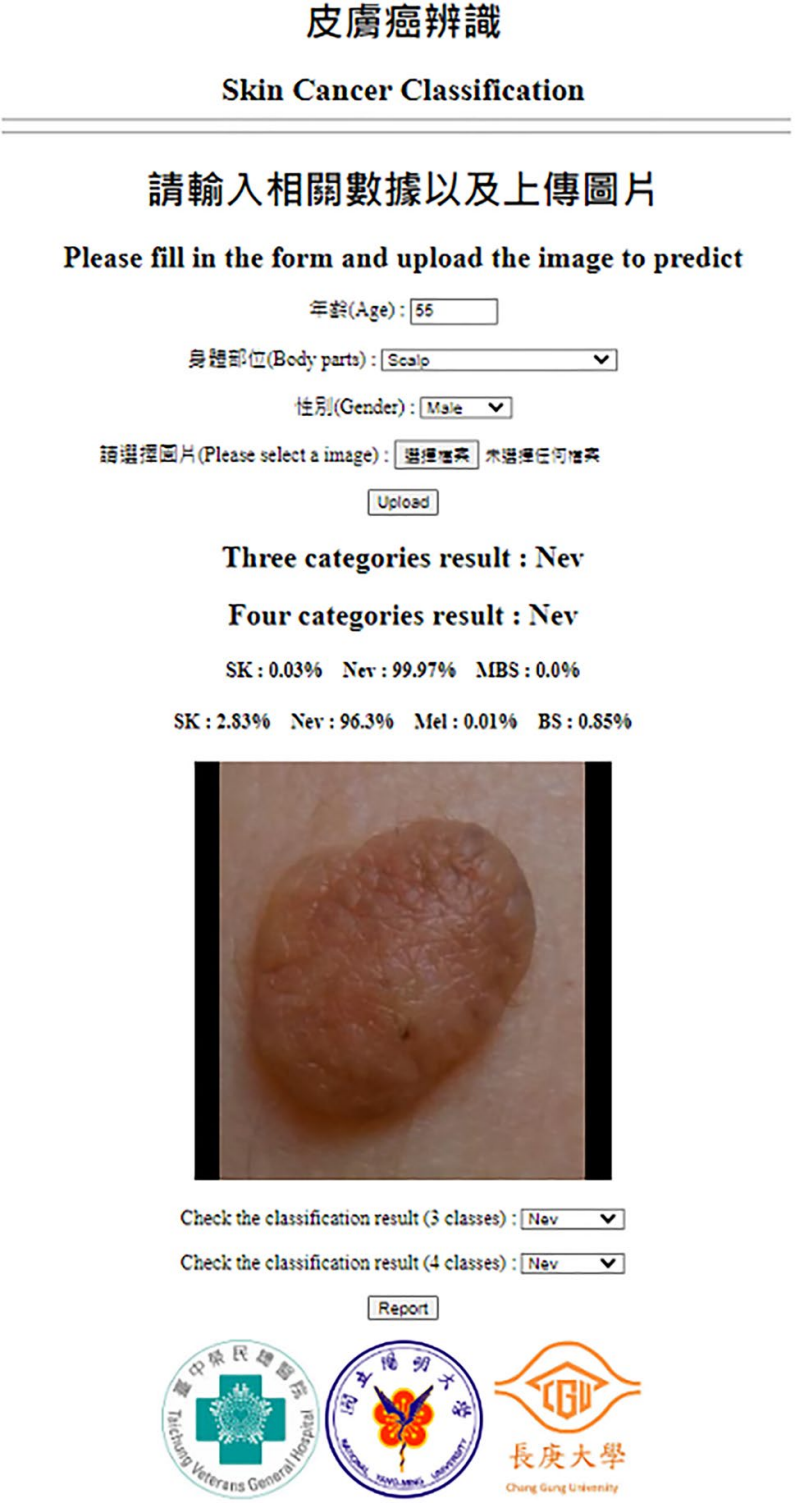
Skin cancer identification system.

To further compare the performance of SkinFLNet with dermatologists, 68 BCC, 12 Mel, 25 SCC, 48 SK, and 122 Nev images were randomly selected from the testing dataset listed in Table 1. Three board-certified dermatologists from the society of the Taiwanese Dermatological Association blindly examined the same images tested for the fusion model of DCNNs, as depicted in Fig. 2. The SkinFLNet fusion model used the pair algorithms of InceptionV3 & ResNet50. The micro-average ROC curves of SkinFLNet for BCC, Mel, Nev, SCC, and SK are shown in Fig. 1. The results demonstrate that SkinFLNet's performance is comparable to, or even better than, that of board-certified dermatologists.

## Discussion

In this study, we presented an efficient skin tumor classification system that combines model fusion and lifelong learning technology. Unlike prior studies that mostly focused on classifying melanoma and benign nevus, our system is capable of efficiently differentiating among five different skin tumor types simultaneously. Moreover, our system achieved satisfactory accuracy in skin tumor diagnosis by utilizing only clinical images, which is comparable to or even outperforms board-certified dermatologists.

Considerable efforts are underway to develop automated image analysis systems that accurately detect diseases. In recent years, DCNNs have become popular for their ability to learn features and classify objects effectively. Esteva et al.^13^ show that a DCNN trained on a large dataset (> 120,000 images) could achieve dermatologist-level classification accuracy in differentiating between melanoma and benign nevi. Similarly, Yang et al.^25^ used a dermatologist criteria-inspired representation to diagnose clinical skin lesions based on the SD-198 dataset. Their proposed method outperformed other deep learning methods but not dermatologists, achieving an accuracy of 57.62%. Moreover, experts in dermatology achieved an average accuracy of 83.29%^25^. Han et al.^19^ classified clinical images of 12 skin diseases using ResNet-152 on three datasets, totaling 19,398 images, and their algorithm performed similarly to 16 dermatologists. However, a lower tested algorithm performance was reported using a relatively limited dataset of 6009 clinical images for 14 diagnoses^26^. In a different study, Brinker et al.^27^ compared the performance of a dermoscopy-trained ResNet50 algorithm with 145 dermatologists for melanoma detection on clinical skin lesion images (MClass-ND). The deep learning method achieved a similar sensitivity and better specificity score than dermatologists. Our study demonstrates that our DCNN-based skin tumor classification systems, using a fusion model and lifelong technologies, can achieve similar performance levels, using almost 50-fold fewer clinical images (< 2000 images for training), without dermoscopic images.

Our approach involves model fusion and combines classification outcomes from paired models to identify the optimal result. The method we propose employs a model fusion-based approach, where we merge the classification results from multiple models and subsequently select the most accurate one. Prior studies have proposed ensemble learning based methods, such as fusion techniques and methods related to boosting techniques^28,29^. The "Cost-sensitive Boosting Pruning Trees" methodology involves feature extraction from diverse data sources^28^. This technique leverages boosting pruning trees to enhance classification accuracy. However, this method is not tailored for image classification. Conversely, "AdaD-FNN" employs a fuzzy stacking approach to enhance features extracted from Chest Computed Tomography (CCT) images, improving classification accuracy^29^. Both these approaches are geared towards augmenting classification accuracy through data feature extraction or enhancement. In contrast, our method places emphasis on model fusion, wherein we integrate classifications from disparate models/algorithms to achieve heightened accuracy. It is noteworthy that the images we work with are colorful, captured using digital cameras and mobile phones, thus differing from the data sources included in the previous studies. Moreover, the algorithms used in their studies are not available as open-source solutions, rendering a direct comparison in our experiments unfeasible.

There are some advantages in the present study. Many studies in this field have primarily examined two essential binary distinctions, such as differentiating between malignant melanoma and benign nevus. Our method can concurrently distinguish among the five types of malignant and benign tumors, and that holds significant relevance in clinical settings. Our contribution also lies in introducing a combinatorial approach, employing multi-model fusion to enhance classification accuracy. This methodology surpasses the performance of renowned image classification algorithms, including but not limited to VGG, ResNet, and Inception. Furthermore, we have embraced a lifelong learning strategy, ensuring the adaptability of our method to real-world clinical practice where symptoms can exhibit variability. This method allows the model to self-update, maintaining its continuous relevance and user-friendliness.

Our study has a few limitations that must be acknowledged. Firstly, the limited number of clinical images used to train our model may have restricted its generalization to a wider population. Therefore, we utilized image augmentation to reduce the bias caused by the imbalance. Additionally, we have deployed our model in real-world clinics to enable lifelong training and improve its accuracy. Secondly, due to the low incidence of melanoma among Asians, we had an insufficient number of cases to validate our model's accuracy. Thirdly, due to its diverse clinical presentations, SK was frequently misclassified as either malignant or other benign conditions. This highlights the limitations of relying solely on clinical images as the input source for our work. Therefore, we have also incorporated demographic covariates such as age, sex, and locations of lesions, which may enhance the model's performance. Our study's strength lies in using the DCNN's output to calculate the probability of malignancy, providing useful information for clinicians in their decision-making process regarding the necessity of a biopsy. While AI systems, including our DCNN, have shown promising results in skin cancer diagnosis, it is crucial to note that a biopsy and histological examination remain the gold standard for confirmation of the algorithm's diagnosis. Presently, the SkinFLNet has been successfully implemented at Taichung Veterans General Hospital in Taichung, Taiwan, as displayed in Fig. 2.

## Conclusion

SkinFLNet has shown promising performance in classifying different skin tumors and even outperformed board-certified dermatologists in some cases. This could be a valuable tool for assisting general practitioners or dermatologists in diagnosing skin tumors and improving accuracy. However, it's important to note that this study was conducted in a specific setting with a relatively limited dataset, so further research is needed to evaluate the generalizability and reliability of SkinFLNet in other populations.

## Methods

### System architecture

Recently, ensemble methods combining multiple deep learning neural networks have been proposed to enhance the performance of physical examination data. Moreover, "lifelong learning" has been adopted to update the trained model with new data, preventing it from becoming frozen. In this study, we propose the SkinFLNet system, which combines two main strategies: model fusion and lifelong learning. Seven convolutional neural network algorithms were used to train the classification models, and the system architecture is depicted in Supplementary Fig. S1.

### Deep neural network models

The SkinFLNet system utilizes seven convolutional neural network algorithms, including VGG16^30^, VGG19, InceptionResNetV2^31^, InceptionV3^32^, ResNet50^33^, MobileNet^34^, and MobileNetV2, to train the classification models. Among these algorithms, ResNet50 is notable for its residual block architecture. In comparison to ordinary network structures, the residual block includes an additional connection between the input and output of the block, which consists of three stacked convolution layers. This connection is known as a shortcut connection, and it directly links the input of the residual block to the output layer. This architecture addresses the degradation issue in deep networks.

InceptionV3 is the third iteration of the GoogLeNet architecture and utilizes the Inception Module. The module is designed to address the issue of overfitting and computational resource consumption caused by a large number of parameters in the network. In Inception Module V2, two 3 × 3 convolutions replaced the 5 × 5 convolutions used in Inception Module V1 to decrease the number of parameters, and Batch Normalization was added to speed up convergence. In Inception Module V3, the Factorization method is introduced, which splits a two-dimensional convolution into two one-dimensional convolutions to decrease the number of parameters.

InceptionResNetV2 is a neural network architecture that utilizes the Inception-ResNet module, Reduction module, and Stem module. The Inception-ResNet module combines the Inception and ResNet architectures and includes three variations: Inception-ResNet-A, Inception-ResNet-B, and Inception-ResNet-C. The Reduction module is designed to reduce the size of the feature map and incorporates parallelism, Factorization, and a 1 × 1 convolution layer to minimize computation. The Stem module is used at the front of the network for feature extraction.

The VGG is a deep network architecture proposed by the Visual Geometry Group, which won first place in the Localization Task and second place in the Classification Task in the 2014 ILSVRC competition. VGG16 consists of 13 convolutional layers and three fully connected layers, whereas VGG19 has 16 convolutional layers and three fully connected layers.

MobileNet is a neural network architecture introduced by Google in 2017 that reduces the computational load of traditional convolutional neural networks through the use of Depthwise Separable Convolution and Pointwise Convolution. The MobileNet network structure consists of 29 layers. In addition to the standard convolutional kernel used in the first layer, the remaining convolutional layers use Depthwise Separable Convolution and Pointwise Convolution. MobileNetV2 further reduces the number of parameters and computation by introducing Inverted Residuals and Linear Bottlenecks to the MobileNet architecture.

### Lifelong learning

Lifelong learning focuses on developing techniques and architectures that enable models to learn sequentially without retraining from scratch. The proposed lifelong learning algorithm is based on transfer learning, where images with classification errors are used as input for transfer learning. To improve the model's classification accuracy, we propose a lifelong learning algorithm that retrains the model with a combined dataset, including newly collected data and part of the original data. The procedure and pseudo code for the proposed lifelong learning algorithm is shown in Supplementary Figs. S2 and S3, respectively.

### Model fusion

The sum of absolute values is obtained by subtracting the predictions of any two different models from the seven models above, as shown in Eq. (1). In this equation, C represents the total number of categories, i represents the category, A and B represent any two of the seven CNN models, PiA represents the probability of class i in model A, and PiB represents the probability of class i in model B. Next, we determine the two CNN models with the largest sum, representing the best complementarity of the two CNN models. We then average their prediction probability, as shown in Eq. (2). The procedure and pseudo code of algorithm are illustrated in Supplementary Figs. S4 and S5.

1 $${argmax}_{A,B}\left(\sum_{i=1}^{C}\left|{P}_{iA}-{P}_{iB}\right|\right)$$

2 $${argmax}_{i}\left(\frac{{P}_{i\widehat{A}}+{P}_{i\widehat{B}}}{2}\right)for i\le C \,and \,i\ge 1$$

### Evaluation methods

To compare different methods, accuracy alone is often insufficient, and multiple other metrics should be used to provide an overall evaluation. For instance, one method may have a high accuracy rate, but the dataset may be imbalanced, with the model being biased toward a particular class that dominates the data. This can lead to the model simply selecting the dominant class as the prediction without actually learning anything about the data. Therefore, other measures such as precision, recall, F1-score, and confusion matrix should also be considered to gain a more comprehensive understanding of the performance of a method.

The metrics used to compare different methods are weight precision (WP), weight recall (WR), weight F-score (WF), weight sensitivity (WS), and weight specificity (WSP). True positive (TP), true negative (TN), false positive (FP), and false negative (FN) are denoted as TP, TN, FP, and FN, respectively. Precision determines the reproducibility of the measurement or the number of predictions that were correctly labeled as positive, $\frac{TP}{TP+FP}$ and weight precision is the weighted mean of precision with weights equal to class probability, as shown in Eq. (3). Recall shows how many positive instances were correctly identified, $\frac{TP}{TP+FN}$ and the weight recall is the weighted mean of recall with weights equal to class probability, as shown in Eq. (4). F-score combines precision and recall to calculate a score that can be interpreted as an average of both, as shown in $2*\frac{\mathrm{Precision}*\mathrm{Recall}}{\mathrm{Precision}+\mathrm{Recall}}$. Weight F-score is the weighted mean of recall with weights equal to class probability, as shown in Eq. (5). Weight sensitivity is the weighted mean of sensitivity with weights equal to class probability, as shown in Eq. (6). Weight specificity is the weighted mean of specificity with weights equal to class probability, as shown in Eq. (7).

3 $$weight \,precision = \mathop \sum \limits_{i = BCC \ldots SK} \left( {\frac{{num\_of\_data_{i} }}{{num\_of\_data_{BCC} + num\_of\_data_{Mel} + num\_of\_data_{Nev} + num\_of\_data_{SCC} + num\_of\_data_{SK} }}*pricision_{i} } \right)$$

4 $$weight \,recall = \mathop \sum \limits_{i = BCC \ldots SK} \left( {\frac{{num\_of\_data_{i} }}{{num\_of\_data_{BCC} + num\_of\_data_{Mel} + num\_of\_data_{Nev} + num\_of\_data_{SCC} + num\_of\_data_{SK} }}*recall_{i} } \right)$$

5 $$weight \,F-score = \frac{2*weight \,precision*weight \,recall}{{\left( {weight \,precision + weight \,recall} \right)}}$$

6 $$weight \,sensitivity = \mathop \sum \limits_{i = BCC \ldots SK} \left( {\frac{{num\_of\_data_{i} }}{{num\_of\_data_{BCC} + num\_of\_data_{Mel} + num\_of\_data_{Nev} + num\_of\_data_{SCC} + num\_of\_data_{SK} }}*sensitivity _{i} } \right)$$

7 $$weight \,specificity=\sum_{i=BCC\dots SK}\left(\frac{{num\_of\_data}_{i}}{{num\_of\_data}_{BCC}+{num\_of\_data}_{Mel}+{num\_of\_data}_{Nev}+{num\_of\_data}_{SCC}+{num\_of\_data}_{SK}}*{specificity }_{i}\right)$$

### IRB approval status

The study was conducted per the Declaration of Helsinki. The need for informed consent has been waived, and approved by the Ethics Committee of Taichung Veterans General Hospital (CE21044A-1) and Taipei Veterans General Hospital (2021-07-021CC).

### Supplementary Information

Supplementary Figures.

## Data Availability

The datasets used and/or analyzed during the current study are available from the corresponding author upon reasonable request.
