# Supplementary material for: Deep convolutional neural network with fusion strategy for skin cancer recognition: model development and validation
Source: Sci Rep. 2023 Oct 10;13:17087. doi: 10.1038/s41598-023-42693-y (PMC10564722; doi:10.1038/s41598-023-42693-y)
Supplement: Supplementary file 1 — Supplementary Figures. [file 41598_2023_42693_MOESM1_ESM.pdf]

**Supplementary Figure 1. The system architecture of SKinFLNet.**

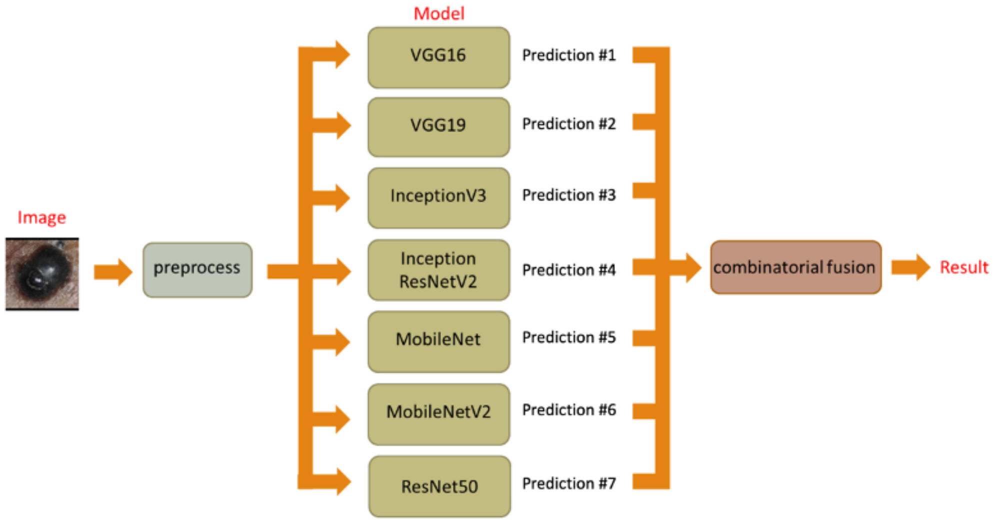

**Supplementary Figure 2. Procedure of Lifelong Learning**

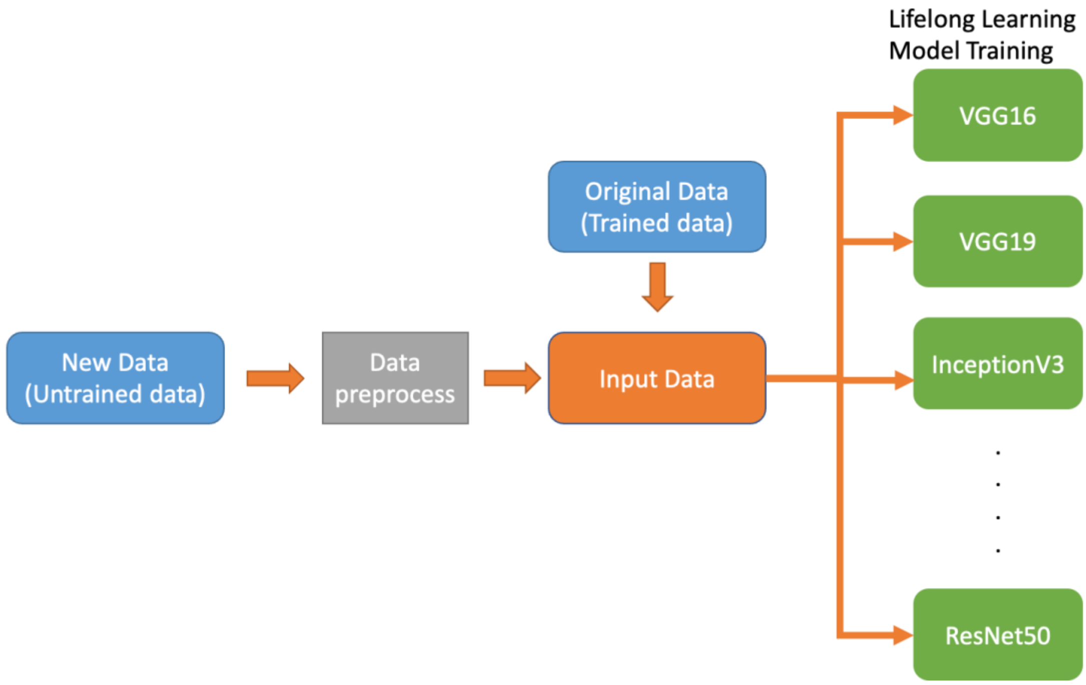

**Supplementary Figure 3. Algorithm of Lifelong Learning**

**Algorithm** Lifelong learning

**require**: 2 CNN models selected after combinatorial fusion ($CNN\_model_{cf}\_list$)
**require**: untrained new dataset ($dataset_{untrained}$)
**require**: original dataset ($dataset_{original}$)
**input**: $dataset_{untrained}$ and $dataset_{original}$
**for** i in $dataset_{untrained}$
    $I_{untrained\_aug} = Rotate(I_i)$
    $I_{untrained\_res} = Resize(I_{untrained\_aug})$
# Augment the images so that the number of images in each category is the same.
$I_{ori\_random} = Random(dataset_{original})$
#Randomly select image from $dataset_{original}$
$I_{train} = Mix(I_{untrained\_res}, I_{ori\_random})$
#Mix $I_{untrained\_res}$ and $I_{ori\_random}$ together in a 1:1 ratio to become a new training dataset
**for** i in $CNN\_model_{cf}\_list$
    **for** epoch = 0,…,$N_{epoch}$ **do**
        $model_{cfi}(I_{train})$
    **end for** convergence
**output**: $model_{continuous\_}list$

**Supplementary Figure 4. Procedure of Combinatorial Fusion of Models for Skin Cancer Classification**

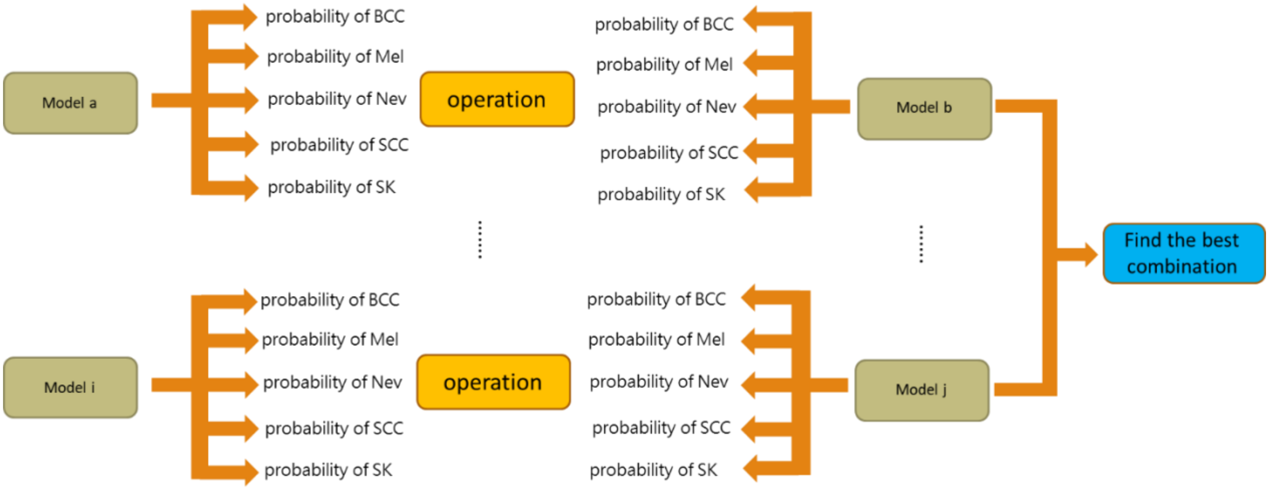

**Supplementary Figure 5. Algorithm of Combinatorial Fusion**

**Algorithm** Combinatorial Fusion

1: **require**: augment and resize images<br>
2: **require**: trained_CNN_model_list [VGG16, VGG19, InceptionResNetV2, InceptionV3, ResNet50, MobileNet, MobileNetV2]<br>
3: **input**: training image ($I_{res}$)<br>
4: **for** i in trained_CNN_model_list<br>
5:     prediction[i] = net.predict($I_{res}$)[0]<br>
6: result = F1(prediction)<br>
7: # The F1 function is the sum of the absolute values obtained by subtracting any two non-repeating values.<br>
8: combination = find_max(result)<br>
9: # Find the combination with the largest 'result' value<br>
10: $Predition_{mean}$= mean(combination)<br>
11: # mean predicted value for this combination<br>
12: **output**: $Predition_{mean}$
